# Un llamado ético a la inclusión de mujeres embarazadas en investigación: Reflexiones del Foro Global de Bioética en Investigación

**DOI:** 10.26633/RPSP.2017.13

**Published:** 2017-02-08

**Authors:** Carla Saenz, Jackeline Alger, Juan Pablo Beca, José M. Belizán, María Luisa Cafferata, Julio Arturo Canario Guzmán, Jesica E. Candanedo P., Lissette Duque, Lester Figueroa, Ana Garcés, Lionel Gresh, Ida Cristina Gubert, Dirce Guilhem, Gabriela Guz, Gustavo Kaltwasser, A. Roxana Lescano, Florencia Luna, Alexandrina A. M. Cardelli, Ignacio Mastroleo, Irene N. Melamed, Agueda Muñoz del Carpio Toia, Ricardo Palacios, Gloria I. Palma, Sofía P. Salas, Xochitl Sandoval, Sergio Surugi de Siqueira, Hans Vásquez, Bertha Ma. Villela de Vega

**Affiliations:** 1 Programa Regional de Bioética Organización Panamericana de la Salud Washington, DC. Estados Unidos de América Programa Regional de Bioética, Organización Panamericana de la Salud, Washington, DC., Estados Unidos de América; 2 Unidad de Investigación Científica, Facultad de Ciencias Médicas Universidad Nacional Autónoma de Honduras (UNAH) Tegucigalpa Honduras Unidad de Investigación Científica, Facultad de Ciencias Médicas, Universidad Nacional Autónoma de Honduras (UNAH), Tegucigalpa, Honduras; 3 Centro de Bioética Facultad de Medicina Clínica Alemana Universidad del Desarrollo Santiago Chile Centro de Bioética Facultad de Medicina Clínica Alemana, Universidad del Desarrollo, Santiago, Chile; 4 Instituto de Efectividad Clínica y Sanitaria (IECS) Instituto de Efectividad Clínica y Sanitaria (IECS) Buenos Aires Argentina Instituto de Efectividad Clínica y Sanitaria (IECS), Buenos Aires, Argentina; 5 Departamento de Salud de Madre y Niño Instituto de Efectividad Clínica y Sanitaria (IECS) Buenos Aires Argentina Departamento de Salud de Madre y Niño, Instituto de Efectividad Clínica y Sanitaria (IECS), Buenos Aires, Argentina; 6 Centro Nacional de Investigaciones en Salud Materno Infantil Dr. Hugo Mendoza (CENISMI) Centro Nacional de Investigaciones en Salud Materno Infantil Dr. Hugo Mendoza (CENISMI) Santo Domingo República Dominicana Centro Nacional de Investigaciones en Salud Materno Infantil Dr. Hugo Mendoza (CENISMI), Santo Domingo, República Dominicana; 7 Ministerio de Salud Ministerio de Salud Ciudad de Panamá República de Panamá Ministerio de Salud, Ciudad de Panamá, República de Panamá; 8 Comisión Nacional de Bioética Quito Ecuador Comisión Nacional de Bioética, Quito, Ecuador; 9 Proyecto Red Global Chimaltenango INCAP Guatemala Guatemala Proyecto Red Global Chimaltenango, INCAP, Guatemala, Guatemala; 10 Instituto de Nutrición de Centroamérica y Panamá Guatemala Guatemala Instituto de Nutrición de Centroamérica y Panamá, Guatemala, Guatemala; 11 Instituto de Ciencias Sostenibles Instituto de Ciencias Sostenibles Managua Nicaragua Instituto de Ciencias Sostenibles, Managua, Nicaragua; 12 Comité de ética en Investigación con Seres Humanos Universidade Federal do Paraná Curitiba Brasil Comité de ética en Investigación con Seres Humanos, Universidade Federal do Paraná, Curitiba, Brasil; 13 Faculdade de Ciências da Saúde Universidade de Brasília Brasil Faculdade de Ciências da Saúde, Universidade de Brasília, Brasilia; 14 Portal da Bioética Portal da Bioética São Paulo Brasil Portal da Bioética, São Paulo; 15 Consultor en ética de la Investigación Biomédica Consultor en ética de la Investigación Biomédica Santiago Chile Consultor en ética de la Investigación Biomédica, Santiago, Chile; 16 Universidad Peruana Cayetano Heredia Universidad Peruana Cayetano Heredia Lima Perú Universidad Peruana Cayetano Heredia, Lima, Perú; 17 Programa de Bioética Facultad Latinoamericana de Ciencias Sociales (FLACSO) Buenos Aires Argentina Programa de Bioética, Facultad Latinoamericana de Ciencias Sociales (FLACSO), Buenos Aires, Argentina; 18 Comité de ética en la Investigación con Seres Humanos Universidad Estatal de Londrina Paraná Brasil Comité de ética en la Investigación con Seres Humanos, Universidad Estatal de Londrina, Paraná, Brasil; 19 CONICET CONICET Buenos Aires Argentina CONICET, Buenos Aires, Argentina; 20 Programa de Bioética Facultad Latinoamericana de Ciencias Sociales (FLACSO) Buenos Aires Argentina Programa de Bioética, Facultad Latinoamericana de Ciencias Sociales (FLACSO), Buenos Aires, Argentina; 21 Universidad Católica de Santa María Universidad Católica de Santa María Arequipa Perú Universidad Católica de Santa María, Arequipa, Perú; 22 División de Ensayos Clínicos y Farmacovigilancia Instituto Butantan São Paulo Brasil División de Ensayos Clínicos y Farmacovigilancia, Instituto Butantan, São Paulo, Brasil; 23 Departamento de Microbiología, Facultad de Salud Universidad del Valle Cali Colombia Departamento de Microbiología, Facultad de Salud, Universidad del Valle, Cali, Colombia; 24 Facultad de Medicina Universidad Diego Portales Santiago Chile Facultad de Medicina, Facultad de Medicina, Santiago, Chile; 25 Investigación Materno Infantil Instituto Nacional de Salud San Salvador El Salvador Investigación Materno Infantil, Instituto Nacional de Salud, San Salvador, El Salvador; 26 Pontificia Universidad Católica de Paraná Pontificia Universidad Católica de Paraná Curitiba Brasil Pontificia Universidad Católica de Paraná, Curitiba, Brasil; 27 Instituto Nacional de Salud Instituto Nacional de Salud Lima Perú Instituto Nacional de Salud, Lima, Perú; 28 Ministerio de Salud Pública y Asistencia Social Ministerio de Salud Pública y Asistencia Social Guatemala Guatemala Ministerio de Salud Pública y Asistencia Social, Guatemala

El Foro Global de Bioética en Investigación (GFBR por sus siglas en inglés) se reunió el 3 y 4 de noviembre en Buenos Aires, Argentina, con el objetivo de discutir la ética de la investigación con mujeres embarazadas. El GFBR es una plataforma mundial que congrega a actores clave con el objetivo de promover la investigación realizada de manera ética, fortalecer la ética de la investigación en salud, particularmente en países de ingresos bajos y medios, y promover colaboración entre países del norte y del sur.^a^ Los participantes en el GFBR provenientes de Latinoamérica incluyeron a eticistas, investigadores, miembros de comités de ética y representantes de autoridades sanitarias provenientes de Argentina, Brasil, Chile, Colombia, Ecuador, El Salvador, Guatemala, Honduras, Panamá, Perú, Nicaragua y la República Dominicana.

Una legítima preocupación por la protección de las mujeres embarazadas y sus embriones o fetos ha llevado a la mayoría de los países de la Región de las Américas a limitar la realización de estudios con mujeres embarazadas exclusivamente a aquellos estudios específicos sobre el embarazo, y a requerir la exclusión sistemática de las mujeres embarazadas o de las mujeres que quedan embarazadas en el curso del estudio. Ciertamente, a lo largo de la historia de la ética de la investigación, se ha creído erróneamente que proteger a una población es sinónimo de excluirla de los estudios. Se sabe ahora que proceder así implica exponer a riesgos mucho mayores a la población que se busca proteger.

El embarazo implica cambios fisiológicos sustantivos e impacta profundamente la manera como el cuerpo metaboliza los medicamentos. Sin embargo, por evitar hacer investigación con mujeres embarazadas, no se ha producido la evidencia científica necesaria para tomar decisiones sobre tratamientos e intervenciones preventivas con dosis eficaces y seguras para ellas y sus embriones o fetos. A manera de ilustración, en el 2001 había en los Estados Unidos apenas más de una docena de medicamentos aprobados para uso en el embarazo (1) y en el 2011 la Food and Drug Administration (FDA) aprobó por primera vez en 15 años un medicamento para su uso en el embarazo (2). Como consecuencia de no haber producido la evidencia necesaria, se pone en riesgo la salud de las mujeres embarazadas cada vez que se les da atención médica. Las mujeres embarazadas se enferman y las mujeres enfermas se embarazan, y no se sabe si los medicamentos que se les da son eficaces o siquiera seguros para ellas y sus embriones o fetos.

Los investigadores, los profesionales de la salud, las autoridades sanitarias, los miembros de los comités de revisión ética, los eticistas y la comunidad científica en general tenemos la obligación moral de revertir esta situación. Tenemos el deber de promover activamente la investigación con mujeres embarazadas, que no solo es permitida por las pautas éticas internacionales sino que además es un imperativo moral. Rehusarse a hacer investigación durante el embarazo es perpetuar el riesgo al que exponemos a millones de mujeres embarazadas diariamente. Se estima que el 94% de las mujeres embarazadas en los Estados Unidos usa por lo menos un medicamento que requiere prescripción, y que alrededor del 50% usa cuatro o más medicamentos durante el embarazo (3). La inclusión responsable de las mujeres embarazadas en la investigación es un tema de equidad y justicia social (4).

Los autores hacemos por ello un llamado a la reflexión sobre la importancia de hacer investigación con mujeres embarazadas y a tomar acciones para promoverla en la Región. Debemos cambiar el paradigma de los investigadores, miembros de comités de revisión ética, patrocinadores y autoridades sanitarias, que excluyen sistemáticamente a las mujeres embarazadas de las investigaciones, a la par que fortalecemos nuestra capacidad para hacer un análisis ético riguroso para determinar, caso por caso, en qué estudios es aceptable incluirlas de manera responsable.

Esta reflexión es oportuna en un contexto en que muchos países latinoamericanos están revisando sus marcos normativos y regulatorios para la investigación con seres humanos, puesto que la inclusión de mujeres embarazadas en la investigación requerirá modificar muchos de los marcos existentes. El brote del virus del Zika ha puesto sobre el tapete la urgencia moral y científica de promover la investigación con mujeres embarazadas (5). La publicación de la revisión de las Pautas éticas Internacionales para la *Investigación Biomédica con Seres Humanos del Consejo de Organizaciones Internacionales de las Ciencias Médicas* (CIOMS por sus siglas en inglés) en diciembre del 2016, que dan orientación sustantiva para incluir a las mujeres embarazadas de manera ética en investigaciones, es un momento oportuno para llamar a la reflexión y al cambio en nuestra Región (6).

## Agradecimiento.

La edición del GFBR del 2016 ha sido financiada por el Wellcome Trust, los Institutos Nacionales de la Salud de los Estados Unidos (NIH, por sus siglas en inglés), el Consejo de Investigación Médica del Reino Unido y la Fundación Bill y Melinda Gates.

## Footnotes

^a^ Más información sobre el GFBR en http://www.gfbr.global/.

## References

[1] 1. Haire D. FDA approved obstetrics drugs: their impact on mother and baby. National Women’s Health Alliance. 2001.

[2] 2. Norwitz ER, Greenberg JA. FDA approval for use of medications in pregnancy: an uphill battle. Rev Obstet Gynecol. 2011;4(2):39–41. PMC3218552 22102925

[3] 3. Mitchell AA, Gilboa SM, Werler MM, Kelley KE, Louik C, Hernández-Díaz S et al. Medication use during pregnancy, with particular focus on prescription drugs: 1976–2008. Am J Obstet Gynecol. 2011;205(1):51–68. 10.1016/j.ajog.2011.02.029 PMC3793635 21514558

[4] 4. Lyerly AD, Little MO, Faden R. The second wave: toward responsible inclusion of pregnant women in research. Int J Fem Approaches Bioeth. 2008; 1(2):5–22. 10.1353/ijf.0.0047 PMC2747530 19774226

[5] 5. Organización Panamericana de la Salud. Consulta de ética sobre el zika: orientación ética sobre cuestiones clave planteadas por el brote. Washington, D.C.: Organización Panamericana de la Salud; 2016. Disponible en: http://iris.paho.org/xmlui/bitstream/handle/123456789/28485/OPSKBR16002_spa.pdf?sequence=6&isAllowed=y Acceso el 20 de diciembre de 2016.

[6] 6. Consejo de Organizaciones Internacionales de las Ciencias Médicas. International Ethical Guidelines for Biomedical Research Involving Human Subjects. Ginebra: Consejo de Organizaciones Internacionales de las Ciencias Médicas; 2016. Disponible en: www.cioms.ch/ethical-guidelines-2016 Acceso el 20 de diciembre de 2016.

